# Supplementary material for: Elucidation of the anti-autophagy mechanism of the Legionella effector RavZ using semisynthetic LC3 proteins
Source: eLife. 2017 Apr 11;6:e23905. doi: 10.7554/eLife.23905 (PMC5388539; doi:10.7554/eLife.23905)
Supplement: Supplementary file 1. — DOI: http://dx.doi.org/10.7554/eLife.23905.019 [file elife-23905-supp1.docx]

**Supplementary file 1.**

**Mass characterization of peptides by HR-MS.**

| **peptide sequence**  **Peptide Sequence**  **(from N to C)** | **MS form** | **M_W_ cal**  **(Da)** | **M_W_ found**  **(Da)** | **∆ M_W_**  **(Da)** |
| --- | --- | --- | --- | --- |
| CQETFG(p) | C_64_H_88_ O_13_N_7_S_2_ [M+H]^+^ | 1226.58760 | 1226.59177 | 0.00417 |
| CQETFG-PE(p) | C_101_H_159_N_8_O_20_PS_2_ [M+H]^+^ | 1900.40459 | 1900.40462 | 0.00003 |
| CQETFG-PE | C_69_H_122_O_18_N_8_PS_2_ [M+H]^+^ | 1445.80506 | 1445.80896 | 0.00390 |
| CQETFG-DHPE | C_49_H_88_O_18_N_8_PS_2_ [M+H]^+^ | 1165.49206 | 1165.49380 | 0.00174 |
| CQETFG-C16(p) | C_80_H_120_O_13_N_7_S_2_ [M+H]^+^ | 1450.83800 | 1450.83936 | 0.00136 |
| CQETFG-C16 | C_48_H_82_O_11_N_7_S_2_ [M+H]^+^ | 996.55082 | 996.55168 | 0.00086 |
| DIDEFDLLEGDE | C_60_H_89_O_27_N_12_ [M+H]^+^ | 1409.59546 | 1409.59665 | 0.00119 |

(p) represents the protected form of the peptides. Side-chain-protecting groups: *t-*Bu for Glu and Thr; Trt for Gln; S*t-*Bu for Cys. N-terminal amide group of the peptides was protected by Boc group. The S*t-*Bu group was kept in the deprotected forms and needed to be removed by reductive reagent (TCEP) when the peptides were used for protein ligation.
