# Supplementary material for: Elucidation of the anti-autophagy mechanism of the Legionella effector RavZ using semisynthetic LC3 proteins
Source: eLife. 2017 Apr 11;6:e23905. doi: 10.7554/eLife.23905 (PMC5388539; doi:10.7554/eLife.23905)
Supplement: Supplementary file 2. — DOI: http://dx.doi.org/10.7554/eLife.23905.020 [file elife-23905-supp2.docx]

**Supplementary file 2. Chemical Synthesis of Compound 8 and 10**

**Synthesis of Fmoc-protected ethanolamine 3**

Ethanolamine **1** (302 uL, 5 mmol) was dissolved in 20 mL water and sodium bicarbonate (NaHCO_3_, 1.260 g, 15 mmol) was added with stirring. The resulting solution was cooled to 0° in the ice bath and N-(9-Fluorenylmethoxycarbonyloxy) succinimide (Fmoc-OSu, 2.528 g, 7.5 mmol) was added slowly as a solution in 20 mL dioxane. The resulting mixture was stirred at 0° for 1 h and allowed to warm to room temperature overnight. After evaporation, purification of the resulting residue by flash column chromatography using CH_2_Cl_2_/MeOH (97:3) as eluent to afford protected compound **3** 1.01 g (Yield 71%).

Compound **3** (1.01 g, 3.56 mmol, 71%)

**HR-MS**: *m/z*: calcd 284.12812 [M+H]^+^, found 284.12814 [M+H]^+^

**^1^H NMR** (500 MHz, DMSO) δ 7.88 (d, *J* = 7.5 Hz, 2H), 7.69 (d, *J* = 7.5 Hz, 2H), 7.41 (t, *J* = 7.4 Hz, 2H), 7.32 (td, *J* = 7.4, 1.0 Hz, 2H), 7.22 (t, *J* = 5.6 Hz, 1H), 4.62 (t, *J* = 5.5 Hz, 1H), 4.28 (d, *J* = 6.8 Hz, 2H), 4.20 (t, *J* = 6.9 Hz, 1H), 3.39 (t, *J* = 6.2 Hz, 2H), 3.33 (s, 1H), 3.05 (q, *J* = 6.1 Hz, 2H).

**^13^C NMR** (126 MHz, DMSO) δ 156.67, 144.38, 141.18, 128.04, 127.49, 125.62, 120.54, 65.76, 60.37, 47.20, 43.50.

**Synthesis of Fmoc-EA-PO-glycerol 7**

The cocktail solution was prepared as follows: isopropylideneglycerol (197 μL, 1.58 mmol) and triethylamine (TEA, 1.18 mL, 8.45 mmol) were dissolved in 5 mL anhydrous acetonitrile. And then the isopropylideneglycerol cocktail was kept at −20°C for 90 min for the sake of reservation([Kore et al., 2012](#_ENREF_23)). Fmoc-protected ethanolamine **3** (300 mg, 1.06 mmol) was dissolved in 10 mL anhydrous acetonitrile, cooled to 0°C in an ice bath and triethylamine (TEA, 442 μL, 3.18 mmol) was subsequently added. After 15 min stirring, phosphorus oxychloride (POCl_3_, 119 μL, 1.27 mmol) was added dropwise. The reaction mixture was stirred for 60 min at 0°C and then the isopropylideneglycerol cocktail was added into the reaction. After stirring another 60 min at 0°C, about 2 mL water was added into the reaction. The reaction mixture was evaporated, and the resulting crude product was purified by preparative HPLC affording 300 mg compound **7** (yield 64.8%).

Compound **7** (300 mg, 0.686 mmol, 64.8%)

**HR-MS**: *m/z*: calcd 438.13123 [M+H]^+^, found 438.13120 [M+H]^+^

**^1^H NMR** (400 MHz, DMSO) δ 7.89 (d, *J* = 7.5 Hz, 2H), 7.70 (d, *J* = 7.4 Hz, 2H), 7.48 (t, *J* = 5.6 Hz, 1H), 7.44 – 7.37 (m, 2H), 7.34 (dt, *J* = 7.4, 3.7 Hz, 2H), 4.29 (d, *J* = 6.7 Hz, 2H), 4.26 – 4.17 (m, 1H), 3.95 – 3.81 (m, 3H), 3.76 (dt, *J* = 10.3, 6.4 Hz, 1H), 3.60 (tt, *J* = 9.7, 4.9 Hz, 1H), 3.34 (dd, *J* = 6.2, 4.9 Hz, 2H), 3.23 (dd, *J* = 11.6, 5.8 Hz, 2H).

**^13^C NMR** (101 MHz, DMSO) δ 156.65, 144.35, 141.19, 128.10, 127.56, 125.67, 120.59, 70.80, 68.09, 65.98, 64.85, 62.74, 47.14, 41.14 .

**^31^P NMR** (162 MHz, DMSO) δ -0.90.

**Synthesis of Fmoc-EA-PO-diacetyl glycerol 9**

Fmoc-EA-PO-glycerol **7** (150 mg, 0.34 mmol) was dissolved in 2 mL acetic anhydride /pyridine = 1/2 (v/v). The reaction mixture was stirred overnight at room temperature. The reaction mixture was co-evaporated with toluene, and the resulting crude product was purified by preparative HPLC, affording 85 mg compound **9** (yield 64.8%).

Compound **9** (85 mg, 0.163 mmol, 45%)

**HR-MS**: *m/z*: calcd 522.15236 [M+H]^+^, found 522.15209 [M+H]^+^

**^1^H NMR** (400 MHz, DMSO) δ 7.89 (d, *J* = 7.5 Hz, 2H), 7.70 (d, *J* = 7.4 Hz, 2H), 7.48 (t, *J* = 5.5 Hz, 1H), 7.42 (t, *J* = 7.3 Hz, 2H), 7.34 (t, *J* = 7.4 Hz, 2H), 5.15 – 5.08 (m, 1H), 4.29 (t, *J* = 11.1 Hz, 2H), 4.25 – 4.19 (m, 2H), 4.18 – 4.09 (m, 1H), 4.06 – 3.96 (m, 2H), 3.86 (dt, *J* = 10.4, 5.3 Hz, 3H), 3.24 (dd, *J* = 11.4, 5.7 Hz, 2H), 2.04 – 1.98 (m, 6H).

**^13^C NMR** (101 MHz, DMSO) δ 170.57, 170.26, 156.65, 144.35, 141.20, 128.09, 127.54, 125.64, 120.59, 70.12, 65.96, 65.22, 64.54, 62.20, 47.15, 41.25, 21.16, 20.94.

**Fmoc deprotection of the compounds**

Fmoc deprotection of Fmoc-EA-PO-glycerol **7** and Fmoc-EA-PO-diacetyl glycerol **9** afforded glycerophosphoethanolamine **8** and diacetyl glycerophosphoethanolamine **10**, respectively. Briefly, 50% diethylamine/dichloromethane solution was added to a solution of Fmoc-EA-PO-glycerol **7** or Fmoc-EA-PO-acetyl glycerol **9**. The reaction was stirred for 5 h and subsequently poured into a flask containing toluene (3 mL). The solvents were removed under reduced pressure, while the temperature was not allowed to exceed 40°C. The solid was solved in toluene again (3 mL) and the solvent was removed under reduced pressure. The product was dried under reduced pressure overnight.
