# Supplementary material for: Elucidation of the anti-autophagy mechanism of the Legionella effector RavZ using semisynthetic LC3 proteins
Source: eLife. 2017 Apr 11;6:e23905. doi: 10.7554/eLife.23905 (PMC5388539; doi:10.7554/eLife.23905)
Supplement: Supplementary file 3. — DOI: http://dx.doi.org/10.7554/eLife.23905.021 [file elife-23905-supp3.docx]

**Supplementary file 3.**

**Mass characterization of modified LC3 proteins by ESI-MS.**

| **proteins** | **C-terminal sequence** | **M_W_ calculated** | | **M_W_ found** | **∆ M_W_** |
| --- | --- | --- | --- | --- | --- |
| MBP-LC3-PE | C^115^QETFG^120^-DPPE | 60080 | 60075 | | 5 |
| LC3-EA | C^115^QETFG^120^-EA | 14366 | 14367 | | 1 |
| LC3-pEA | C^115^QETFG^120^-pEA | 14446 | 14447 | | 1 |
| LC3-GpEA | C^115^QETFG^120^-GpEA | 14520 | 14523 | | 3 |
| LC3-DAGpEA | C^115^QETFG^120^-DAGpEA | 14604 | 14605 | | 1 |
| MBP-LC3-DHPE(6:0) | C^115^QETFG^120^-DHPE | 59800 | 59800 | | 0 |
| MBP-LC3-C16 | C^115^QETFG^120^-C16 | 59630 | 59624 | | 6 |

The ligated proteins were characterized by electrospray ionisation mass spectrometry (ESI-MS). Data evaluation was carried out using the Xcalibur software package and MagTran software program was used for deconvolution of ESI mass spectra.
