# Supplementary material for: Elucidation of the anti-autophagy mechanism of the Legionella effector RavZ using semisynthetic LC3 proteins
Source: eLife. 2017 Apr 11;6:e23905. doi: 10.7554/eLife.23905 (PMC5388539; doi:10.7554/eLife.23905)
Supplement: Supplementary file 4. — DOI: http://dx.doi.org/10.7554/eLife.23905.022 [file elife-23905-supp4.doc]

**Supplementary file 4. Data collection and refinement statistics**

|  | **RavZ^1-431^/LC3B** | **RavZLIR2-LC3B**  **(low-salt)** | **RavZLIR2-LC3B**  **(high-salt)** | **RavZ^1-487^** | **RavZ^20-502^** |
| --- | --- | --- | --- | --- | --- |
| **Data collection** |  |  |  |  |  |
| Space group | P 2_1_ | P 4_3_ 2_1_ 2 | P 4_3_ 2_1_ 2 | I 4 2 2 | I 4 2 2 |
| Cell dimensions |  |  |  |  |  |
| *a*, *b*, *c* (Å) | 51.55, 69.55, 90.17 | 69.61, 69.61, 117.26 | 69.30, 69.30, 117.78 | 221.27, 221.27, 73.27 | 222.77, 222.77, 72.94 |
| α, β, γ (°) | 90.00, 101.08, 90.00 | 90.00, 90.00, 90.00 | 90.00, 90.00, 90.00 | 90.00, 90.00, 90.00 | 90.00, 90.00, 90.00 |
| Resolution (Å) | 48.08-2.47 (2.56-2.47)^a^ | 49.22-1.53 (1.59-1.53) | 49.00-1.90 (1.97-1.90) | 47.05-2.85 (2.95-2.85) | 47.15-2.80 (2.90-2.80) |
| *R*_merge_ | 0.18 (0.72) | 0.18 (2.21) | 0.20 (1.55) | 0.22 (1.97) | 0.19 (1.95) |
| *I* / σ*I* | 9.51 (2.57) | 14.69 (1.42) | 14.37 (1.52) | 14.09 (1.85) | 15.14 (1.94) |
| *CC*_1/2_ | 99.20 (56.80) | 99.90 (17.5) | 99.90 (20.60) | 99.90 (13.00) | 99.80 (17.50) |
| Completeness (%) | 99.92 (99.42) | 100 (100) | 100 (100) | 100 (100) | 100 (100) |
| Redundancy | 6.80 (6.50) | 25.40 (24.60) | 23.90 (15.20) | 26.70 (27.90) | 26.70 (26.30) |
| **Refinement** |  |  |  |  |  |
| Resolution (Å) | 48.08-2.47 | 49.22-1.53 | 49.00-1.90 | 47.05 - 2.85 | 47.15-2.80 |
| No. reflections | 22,607 | 44,237 | 23,348 | 21,565 | 22,915 |
| *R*_work_ / *R*_free_ | 17.74 / 23.84 | 20.96 / 24.25 | 20.82 /26.09 | 20.12/ 24.95 | 20.68 / 25.35 |
| No. atoms |  |  |  |  |  |
| Protein | 4126 | 2110 | 2088 | 2967 | 2908 |
| Ligand/ion | - | 26 | - | 9 | 20 |
| Water | 111 | 198 | 187 | 19 | 14 |
| *B*-factors |  |  |  |  |  |
| Protein | 50.60 | 31.80 | 41 | 115.80 | 114.50 |
| Ligand/ion | - | 44.70 | - | 181 | 159.80 |
| Water | 35.50 | 36.60 | 44 | 78.80 | 85.10 |
| R.m.s deviations |  |  |  |  |  |
| Bond lengths (Å) | 0.010 | 0.008 | 0.008 | 0.009 | 0.009 |
| Bond angles (°) | 1.45 | 1.18 | 1.17 | 1.45 | 1.38 |
| **Validation and Deposition** |  |  |  |  |  |
| Ramachandran plot (%) |  |  |  |  |  |
| Favored region | 97.30 | 99.60 | 100 | 94.61 | 95.88 |
| Allowed region | 2.70 | 0.40 | 0 | 5.39 | 3.85 |
| Outlier region | 0 | 0 | 0 | 0 | 0.27 |
| PDB-ID | 5MS2 | 5MS5 | 5MS6 | 5MS8 | 5MS7 |

Each data set was collected from a single crystal.

Data set of RavZ LIR2-LC3 crystals were collected from two crystallization conditions, as indicated with low-salt and high-salt.

^a^Values in parentheses are for highest-resolution shell.
